# Sintomas Depressivos estão Associados a Níveis Séricos Elevados de Colesterol de Lipoproteína de Baixa Densidade em Idosos com Diabetes Mellitus Tipo 2

**DOI:** 10.36660/abc.20190404

**Published:** 2020-09-18

**Authors:** Etiene Oliveira da Silva Fittipaldi, Armele Dornelas de Andrade, Ana Célia Oliveira Santos, Shirley Campos, Juliana Fernandes, Maria Teresa Jansen de Almeida Catanho

**Affiliations:** 1 Universidade Federal de Pernambuco Recife PE Brasil Universidade Federal de Pernambuco, Recife, PE – Brasil; 2 Universidade de Pernambuco Recife PE Brasil Universidade de Pernambuco, Recife, PE-Brasil

**Keywords:** Doenças Cardiovasculares, Diabetes Mellitus, Hipertensão, Dislipidemias, Depressão, Transtorno Depressivo, Lipoproteinas LDL

## Abstract

**Fundamento:**

O Diabetes Mellitus Tipo 2 (DMT2) é comum nos idosos, que também apresentam um nível elevado de fatores de risco para doenças cardiovasculares (DCVs), tais como dislipidemia. Entretanto, o papel da depressão nos pacientes com DMT2 e sua relação com fatores de risco para DCV são pouco estudados.

**Objetivo:**

O objetivo do presente estudo foi investigar a relação entre sintomas depressivos (SDs) e fatores de risco cardiovascular conhecidos em idosos comunitários portadores de DMT2.

**Métodos:**

Trata-se de um estudo transversal, no qual foram incluídos 85 idosos comunitários com DMT2. Os SDs foram avaliados através da Escala de Depressão Geriátrica de Yesavage, em versão reduzida (GDS-15). Os seguintes fatores de risco cardiovascular foram avaliados: pressão arterial sistólica (PAS) e diastólica (PAD), glicose plasmática em jejum (GPJ), perfil lipídico (triglicerídeos séricos (TG), colesterol total sérico (CT), colesterol sérico de lipoproteína de baixa densidade (LDL-C) e colesterol sérico de lipoproteína de baixa densidade (HDL-C)) e índice de massa corporal (IMC). A análise de regressão múltipla de Poisson foi utilizada para avaliar a associação entre os SDs e cada fator de risco cardiovascular ajustado por sexo, idade, tempo em atividades físicas moderadas e status funcional. O nível de significância adotado para a análise foi de 5%.

**Resultados:**

Dentre todos os fatores de risco analisados, apenas o aumento de LDL-C apresentou uma correlação com níveis elevados de SD (RP=1,005; IC95% 1,002-1,008). Foi observada uma associação significativa entre os níveis de HDL-C (RP=0,99; IC95% 0,98-0,99) e a PAS (RP=1,009; IC95% 1,004-1,014).

**Conclusão:**

Nos idosos com DMT2, a presença de SD foi associada a níveis de LDL-C, HDL-C e PAS, mesmo após o ajuste por sexo, idade, nível de atividade física e capacidade funcional. (Arq Bras Cardiol. 2020; 115(3):462-467)

## Introdução

O Diabetes Mellitus, principalmente do Tipo 2 (DMT2), é uma doença prevalente na população idosa e, muitas vezes, vem acompanhada de comorbidades e síndromes geriátricas, inclusive doenças relacionadas à saúde mental, tais como a depressão.^1-5^ Evidências anteriores mostram que a prevalência de depressão é quase duas vezes maior nas pessoas com diabetes e que a depressão pode aumentar o risco de desenvolvimento de DMT2.^6,7^ Nos idosos com diabetes que vivem em países de baixa e média renda, como o Brasil, essa prevalência pode chegar a 35,7%.^8^

Além disso, os idosos com DMT2 apresentam aumento nos fatores de risco para doença cardiovascular (DCV), tais como dislipidemia, pressão alta, controle glicêmico prejudicado e obesidade.^9,10^ Entretanto, uma definição clara do papel do DMT2 na DCV pode incluir outros fatores psicológicos, tais como a depressão que, combinada com o diabetes, pode aumentar esse risco. Achados anteriores indicam uma relação entre a presença de depressão e, sobretudo, anormalidades no perfil lipídico dos adultos e/ou idosos.^11-14^

Portanto, a depressão e a DCV são comuns em pacientes com DMT2 e apresentam uma correlação. No entanto, a relação entre os sintomas depressivos (SDs) e a DCV nos adultos com DMT2 é pouco estudada e, muitas vezes, sub-reconhecida.^15^ Assim sendo, nosso objetivo é examinar a relação entre os sintomas depressivos e fatores de risco cardiovascular conhecidos, em idosos moradores de comunidades portadores de DMT2.

## Métodos

### Amostra e Desenho do Estudo

Este é um estudo transversal realizado em uma amostra de idosos comunitários da cidade de Recife. O projeto foi aprovado pelo Comitê Institucional de Ética em Pesquisa em Seres Humanos da Universidade de Pernambuco. Todos os participantes que concordaram em participar deste estudo assinaram o termo de consentimento livre e esclarecido.

Os participantes do estudo foram selecionados por conveniência a partir de 3.271 registros médicos de pacientes idosos (60 anos ou mais) com DMT2, que foram atendidos na Clínica de Geriatria e Endocrinologia de uma universidade pública de Recife. Destes pacientes, 871 haviam sido diagnosticados com DMT2 há mais de 2 anos. Esses indivíduos foram contatados por telefone e convidados a participarem da pesquisa. Assim sendo, 198 participantes retornaram o contato e se voluntariaram a participar do estudo, e foram, então, encaminhados para triagem, para verificação de elegibilidade.

Os critérios de inclusão foram: ter recebido diagnóstico médico de DMT2 há pelo menos 2 anos, idade maior ou igual a 60 anos, de ambos os sexos. Os critérios de exclusão foram: indivíduos em uso de insulina, com comprometimento cognitivo, sequelas neurológicas, acuidade visual e/ou auditiva gravemente diminuídas, dores articulares e/ou musculares, amputações de membros inferiores, uso de próteses e/ou limitações físicas impeditivas de locomoção.

Após a aplicação dos critérios de elegibilidade, a amostra foi reduzida a 122 indivíduos. Destes, 37 pacientes se recusaram a fazer o exame de sangue, chegando-se a uma amostra final de 85 idosos com DMT2.

### Medidas

Foram registrados os dados sociodemográficos (idade e sexo), clínicos (sintomatologia depressiva, capacidade funcional e nível de atividade física), bioquímicos e as mensurações antropométricas.

### Sintomas Depressivos

Os sintomas depressivos foram avaliados através da Escala de Depressão Geriátrica de Yesavage, em versão reduzida (GDS-15), que é validada para idosos brasileiros. O escore total varia de 0 a 15 pontos, sendo que escores maiores estão relacionados à presença de sintomas depressivos.^16^ A presença de sintomas depressivos foi dicotomizada quando os participantes tiveram pontuação ≥ 5;^16^ esta categorização foi utilizada apenas para fins descritivos. O escore total foi utilizado na análise multivariada.

### Avaliação das Atividades Instrumentais da Vida Diária

A avaliação da capacidade funcional foi realizada quantitativamente com base nos escores obtidos a partir da Avaliação das Atividades Instrumentais da Vida Diária (AIVD).^17^ A presença de declínio funcional foi observada naqueles pacientes que apresentaram dependência total ou parcial na AIVD.

### Nível de Atividade Física

O nível de atividade física foi obtido com base no tempo em atividade física moderada (T_AFM_) autorrelatado. Esta medida foi calculada a partir das perguntas: “*Durante os últimos 7 dias, em quantos dias você realizou atividades físicas moderadas por pelo menos 10 minutos contínuos?” e “Quanto tempo você geralmente gasta realizando atividades físicas moderadas em um daqueles dias?*”, do Questionário Internacional de Atividade Física. O T_AFM_ foi calculado como se segue = (*n dias*) x (*tempo em min*).^18^

### Doenças Crônicas e Medicações

A presença de outras doenças crônicas além do DMT2 foi registrada como autorrelato pelo entrevistador, através da pergunta “Você já recebeu um diagnóstico médico de outra doença crônica?”. Caso a resposta fosse afirmativa, o entrevistador perguntava detalhes sobre a doença. Todos os pacientes vinham recebendo tratamento farmacológico otimizado para DMT2 por mais de três meses. O medicamento utilizado pelos idosos (o agente hipoglicêmico metformina) era fornecido mensalmente pelo Sistema Único de Saúde (SUS) durante as consultas médicas. Outros medicamentos em uso foram registrados.

### Fatores de Risco Cardiovascular

A glicemia plasmática em jejum (GPJ) e o perfil lipídico (triglicerídeos séricos (TG), colesterol total sérico (CT), colesterol sérico de lipoproteína de baixa densidade (LDL-C) e colesterol sérico de lipoproteína de baixa densidade (HDL-C)) foram coletados a partir de amostras de sangue venoso. A amostra de sangue foi coletada de uma veia na região antecubital, no período da manhã, após jejum de 12 horas, e analisada por um laboratório bioquímico. As análises foram realizadas no Centro Integrado de Saúde Amaury de Medeiros da Universidade de Pernambuco (Cisam/UPE). Os parâmetros medidos incluíram a realização de bioquímicas séricas por meio de um método enzimático automatizado, submetidas aos procedimentos laboratoriais de rotina. O LDL-C foi estimado pela fórmula de Friedewald.^19^ Os valores normais de referência de GPJ, TG, CT, LDL-C, e HDL-C utilizados nesta pesquisa foram definidos pelo Programa Nacional de Educação do Colesterol-Painel de Tratamento do Adulto III (NCEP-ATP III).^20^

A altura (cm) foi medida utilizando-se uma fita métrica fixada à parede com aproximação para 0,1 cm. Para a aferição do peso corporal (kg), foi utilizada uma balança previamente calibrada, com os participantes vestindo roupas leves e descalços. O índice de massa corporal foi calculado considerando o peso e a altura (kg/m^2^).^2^ A avaliação clínica da pressão arterial foi realizada com o paciente sentado, pelo método indireto com técnica auscultatória pelo uso de esfigmomanômetro aneroide.

### Análise Estatística

As variáveis contínuas estão expressas como como média e desvio padrão, e as variáveis categóricas, como valores absolutos e porcentagens. A normalidade dos dados foi testada por meio do teste de Kolmogorov-Smirnov. O gráfico de probabilidade normal e o histograma dos escores dos sintomas depressivos revelaram uma linha curvilínea e distribuição assimétrica positiva. Consequentemente, os escores dos sintomas depressivos foram transformados em log10, a fim de melhorar a linearidade e normalidade. As associações entre a presença de sintomas depressivos e as variáveis categóricas foram testadas através do teste qui-quadrado. Para a análise das variáveis contínuas entre os grupos, foi utilizado o Teste t de Student para amostras independentes.

A regressão múltipla de Poisson foi utilizada para testar a associação entre os sintomas depressivos e cada fator de risco para DCV. Os cálculos da razão de prevalência (RP) foram feitos com intervalo de confiança de 95% (IC95%). Para evitar problemas de multicolinearidade, cada fator de risco cardiovascular e os sintomas depressivos foram analisados separadamente por meio de modelos diferentes. Todos os modelos foram ajustados por sexo, idade, tempo em atividade física moderada e status funcional. Essas covariáveis foram selecionadas tendo como base a revisão de literatura anterior.^21^ O nível de significância adotado para a análise foi 5%. A análise estatística foi realizada com o programa SPSS versão 20.0.

## Resultados

As características da amostra estão expressas na Tabela 1. Vinte e quarto dos oitenta e cinco participantes (28,2%) apresentaram sintomas depressivos. Não houve diferenças entre o sexo e a idade dentre aqueles participantes com e sem sintomatologia depressiva. Entretanto, aqueles participantes com sintomas depressivos tiveram pontuação mais baixa na AIVD (23,63 ± 3,53; valor de p= 0,02) e relataram menos tempo em atividades físicas moderadas (28,63 ± 49,20; valor de p=0,02) quando comparados com os participantes sem sintomas depressivos (Tabela 1).

**Tabela 1:** – Características gerais da amostra de acordo com a presença de sintomas depressivos

	Sintomas depressivos			
Variáveis	Todos	Sim	Não	Valor de p
	(n=85)	(n=61)	(n=24)	
Idade (anos), média ± DP	70,59 (7,43)	69,33 (6,69)	71,08 (7,70)	0,33
Sexo, n (%)				
*Masculino*	20 (23,5)	15 (25,0)	5 (20,8)	0,71
*Feminino*	65 (76,5)	46 (75,4)	19 (79,2)	
Saúde auoavaliada, n (%)				
*Razoável/Ruim*	76 (89,4)	53 (86,9)	23 (95,8)	0,43
*Excelente/Boa*	9 (10,6)	1 (4,2)	8 (13,3)	
Escore AIVD	24,99 (2,85)	25,52 (2,37)	23,63 (3,53)	0,02
IMC (kg/m^2^)^a^	28,63 (5,05)	28,90 (4,81)	27,96 (5,66)	0,45
Atividade física	48,22 (60,46)	56,78 (64,89)	28,63 (42,90)	0,03
Glicemia plasmática em jejum (mg/dL)	159,01 (65,87)	153,75 (63,33)	172,38 (71,57)	0,24
Pressão arterial sistólica (mmHg)^d^	153,06 (80,85)	146,52 (25,69)	142,09 (20,41)	0,37
Pressão arterial diastólica (mmHg)^d^	84,32 (15,83)	82,78 (19,72)	85,17 (14,17)	0,58
HDL-C (mg/dL)	58,53 (23,53)	61,25 (21,75)	52,83 (26,93)	0,16
LDL-C (mg/dL)	106,74 (41,35)	100,18 (33,13)	123,13 (54,31)	0,06
C-total (mg/dL)	194,74 (47,65)	202,88 (58,22)	191,54 (42,92)	0,39
Triglicerídeos (mg/dL)	153,88 (80,85)	151,83 (87,82)	156,83 (62,86)	0,83

*AIVD: Atividades Instrumentais da Vida Diária; IMC: Índice de Massa Corporal;* ^*a*^ *4 valores ausentes;* ^*b*^ *1 valor ausente;* ^*c*^ *6 valores ausentes;* ^*d*^ *3 valores ausentes.*

As doenças crônicas mais frequentes relatadas na nossa amostra foram: hipertensão 10 (11,8%), seguida de osteoartrose 4 (4,7%). Além disso, foram relatadas outras doenças, tais como osteoporose 1 (1,2%), bronquite 1 (1,2%) e hipotireoidismo 1 (1,2%). Os participantes com diagnóstico de hipertensão estavam em uso de inibidores da ECA (enzima conversora de angiotensina), betabloqueadores ou diuréticos.

A Tabela 2 mostra os resultados da associação entre os sintomas depressivos e cada fator de risco para DCV obtidos pelo método de regressão de Poisson. Após ajustes por sexo, idade, atividade física e capacidade funcional, altos níveis de LDL-C foram associados ao aumento dos SDs (RP=1,005; IC95% 1,002-1,008). Níveis de HDL-C e níveis baixos de SD também foram observados (RP=0,99; IC95% 0,98-0,99). Adicionalmente, níveis mais elevados de pressão arterial sistólica foram associados a níveis mais altos de SD (RP=1,009; IC95% 1,004-1,014) (Tabela 2).

**Tabela 2:** – Regressão múltipla de Poisson entre os escores dos sintomas depressivos e fatores de risco cardiovascular

	Sintomas depressivos		
Fatores de risco cardiovascular	RP	IC 95%	valor de p
IMC	0,98	0,96-1,01	0,47
Glicemia plasmática em jejum (mg/dL)	1,001	0,99-1,00	0,32
Pressão arterial sistólica (mmhg)	1,009	1,004-1,014	<0,01
Pressão arterial diastólica (mmHg)	1,00	0,99-1,00	0,42
HDL-C (mg/dL)	0,99	0,98-0,99	<0,01
LDL-C (mg/dL)	1,005	1,002-1,008	<0,01
Triglicerídeos (mg/dL)	1,00	0,99-1,00	0,61
C-total (mg/dL)	1,00	0,99-1,00	0,19

*RP: Razão de prevalência, ajustada por sexo, idade, tempo em atividade física moderada e status funcional.*

## Discussão

Neste estudo, observamos que os níveis de LDL-C, HDL-C e pressão arterial sistólica estavam diretamente relacionados a maiores sintomas depressivos nos idosos com DMT2. Essa associação foi independente do sexo, idade, atividade física e capacidade funcional.

Em estudos anteriores com população geral, os sintomas depressivos foram associados a fatores de risco para DCV^14,22-24^ Entretanto, estudos sobre a associação entre o DMT2 e sintomas depressivos em idosos são raros. Rice et al. (2010) verificaram que níveis elevados de sintomas depressivos em idosas saudáveis estavam associados a níveis mais altos de LDL-C, o que está em consonância com os nossos resultados.^22^ Do mesmo modo, Liang et al. (2014) verificaram que o aumento de sintomas depressivos em idosos chineses estava associado a níveis elevados de LDL-C.^14^ Em uma amostra de 1.469 idosos brasileiros moradores de comunidades, participantes do estudo Bambuí, foi observada uma correlação entre anormalidades no perfil lipídico e sintomas depressivos elevados.^24^

Uma revisão sistemática recente com meta-análise descobriu que taxas menores de depressão estavam relacionadas a níveis séricos mais baixos de LDL-C, quando este foi analisado como medida categórica. No entanto, contrariamente aos nossos achados, níveis baixos de LDL-C estavam presentes em pacientes depressivos, quando essa variável foi tratada como medida contínua.^13^ Os autores discutem uma associação temporal entre o LDL-C sérico e a depressão. Nesta associação, níveis baixos de LDL-C podem estar relacionados com o surgimento da depressão. Por sua vez, os níveis elevados de LDL poderiam ser uma consequência da depressão crônica, na medida em que a progressão da doença poderia acarretar o aumento de peso, e o posterior surgimento da síndrome metabólica e do aumento dos níveis séricos de LDL-C.^13^

A relação entre aqueles fatores de risco e os sintomas depressivos ainda é controversa; alguns estudos desenvolvidos na população idosa em geral verificaram uma associação entre os sintomas depressivos e fatores de risco para DCV^22,25^ ao passo que outros estudos não encontraram correlação alguma entre eles.^26^ Também verificamos uma associação significativa entre outros fatores de risco conhecidos para DCV, tais como pressão arterial alta, níveis elevados de HDL-C e baixos sintomas depressivos. Em um estudo conduzido em adultos latino-americanos com diabetes, a presença de sintomas depressivos foi um fator de risco para a síndrome metabólica, baixos níveis de HDL-C e níveis elevados de triglicerídeos.^23^ A relação entre o aumento da pressão arterial sistólica e sintomas depressivos mais elevados também foi observada em estudos anteriores^27,28^

Algumas vias se conectam na relação entre depressão e desregulações cardiometabólicas. Estudos anteriores demonstraram que estilos de vida pouco saudáveis e a presença de citocinas inflamatórias poderiam estar envolvidos nessa relação.^29-31^ Por exemplo, os indivíduos deprimidos apresentam aumento no consumo de cigarro e álcool. Adicionalmente, esses indivíduos, muitas vezes, têm uma dieta pouco saudável e praticam menos atividade física.^29^Além disso, a depressão pode estar relacionada ao ganho de peso (em parte, resultante da inatividade física e de hábitos alimentares pouco saudáveis), que acarreta inflamação ^29^e está associado à diminuição dos níveis de HDL-C e ao aumento dos níveis de LDL-C.^30^ A associação entre depressão e anormalidades metabólicas pode ser bidirecional, e essa coocorrência pode ser particularmente importante nos pacientes diabéticos. De fato, há evidências de que não é a depressão em si, mas a coocorrência de sintomas depressivos elevados e de anormalidades cardiometabólicas que estão relacionadas ao aumento do risco de diabetes nos idosos.^32^

É importante destacar a influência que algumas medicações ou condições clínicas podem ter sobre a relação entre a sintomatologia depressiva e o risco cardiovascular nos idosos. Observou-se, por exemplo, que algumas medicações, tais como antidepressivos (ex. fluoxetina), podem causar anormalidades lipídicas.^33^ Contudo, na nossa amostra, nenhum dos participantes vinha sendo tratado com essas medicações. Portanto, nossos resultados não foram influenciados por esta variável. Além disso, algumas condições clínicas podem aumentar a presença de sintomas depressivos ou os níveis de colesterol, como no caso dos idosos com hipotireoidismo não diagnosticado ou não tratado.^34^ Entretanto, na nossa amostra, apenas um participante tinha o diagnóstico médico de hipotireoidismo, e essa doença estava controlada com medicação.

Este estudo apresenta algumas limitações que devem ser abordadas. Em primeiro lugar, a amostra de conveniência pequena dificulta a generalização dos resultados e o ajuste de outros fatores de confundimento (como tabagismo, história de abuso de álcool ou uso de medicamento)^29^ pelo método de regressão de Poisson. Além disso, a natureza transversal de nosso estudo não permite determinar se os sintomas depressivos precederam ou sucederam os desfechos CV. Finalmente, as medições da sintomatologia depressiva foram obtidas por meio de autorrelato, utilizando-se o GDS-15, que é considerado uma ferramenta de triagem, e não de diagnóstico clínico. No entanto, ele é um instrumento amplamente utilizado para identificar indivíduos com elevado risco de depressão.^16,35^

Embora haja limitações, até onde sabemos, este é o primeiro estudo a investigar a relação entre sintomas depressivos e fatores de risco cardiovascular em uma amostra de idosos com diabetes. Apesar da pequena magnitude dos nossos resultados, nossos achados podem proporcionar percepções úteis sobre a importância de se avaliar concomitantemente a saúde psicológica e cardiovascular nos idosos diabéticos. Assim sendo, nesse grupo pode ser importante focar em intervenções de estilo de vida e na saúde mental, a fim de verificar se o tratamento da depressão resultará em melhores níveis de colesterol LDL, prevenindo, assim, os desfechos adversos para a saúde. Ademais, pesquisas futuras devem considerar um desenho longitudinal para investigar o surgimento da sintomatologia depressiva nesta população.

## Conclusão

Em uma amostra de idosos com DMT2, a presença de sintomas depressivos foi associada a níveis de LDL-C, HDL-C e pressão arterial sistólica, mesmo após o ajuste por sexo, idade, nível de atividade física e capacidade funcional. Outros estudos são necessários para estabelecer uma relação temporal entre essas duas condições nas populações idosas com Diabetes do Tipo 2.
