# Supplementary material for: Sintomas Depressivos estão Associados a Níveis Séricos Elevados de Colesterol de Lipoproteína de Baixa Densidade em Idosos com Diabetes Mellitus Tipo 2
Source: Arq Bras Cardiol. 2020 Sep 18;115(3):462–7. [Article in Portuguese] doi: 10.36660/abc.20190404 (PMC9363090; doi:10.36660/abc.20190404)
Supplement: Translation

# Translation: Depressive Symptoms are Associated with High Levels of Serum Low-Density Lipoprotein Cholesterol in Older Adults with Type 2 Diabetes Mellitus

**Authors:** Etiene Oliveira da Silva Fittipaldi, Armele Dornelas de Andrade, Ana Célia Oliveira Santos, Shirley Campos, Juliana Fernandes, Maria Teresa Jansen de Almeida Catanho

**Affiliations:** 1 Brazil Universidade Federal de Pernambuco, Recife, PE – Brazil; 2 Brazil Universidade de Pernambuco, Recife, PE-Brazil

**Keywords:** Cardiovascular Diseases, Diabetes Mellitus, Hypertension, Dyslipidemias, Depression, Depressive Disorder, Lipoproteins, LDL

## Abstract

**Background:**

Type 2 Diabetes Mellitus (T2DM) is common in older adults, who also present a high level of risk factors for cardiovascular disease (CVD), such as dyslipidemia. However, the role of depression in T2DM patients and its relationship with CVD risk factors are understudied.

**Objective:**

The present study aimed to investigate the relationship between depressive symptoms (DS) and known cardiovascular risk factors in community dwelling older adults with T2DM.

**Methods:**

This is a cross sectional study, in which 85 community-dwelling older adults with T2DM were assessed. DS was assessed using the Yesavage Geriatric Depression Scale - short version (GDS-15). The following cardiovascular risk factors were evaluated: systolic (SBP) and diastolic blood pressure (DBP), fasting plasma glucose (FPG), lipid profile (serum triglycerides - TG, serum total cholesterol - TC, serum low-density lipoprotein cholesterol - LDL-C, and serum high-density lipoprotein cholesterol - HDL-C) and body mass index (BMI). Poisson multiple regression was performed to test the association between DS and each cardiovascular risk factor adjusted by sex, age, time spent in moderate physical activity, and functional status. The significance level adopted for the analysis was 5%.

**Results:**

Among all the analyzed risk factors, only high levels of LDL-C were related to high DS (PR=1.005, CI 95% 1.002-1.008). A significant association was observed between HDL-C levels (PR=0.99, CI 95% 0.98-0.99) and SBP (PR=1.009, CI 95% 1.004-1.014).

**Conclusion:**

In older adults with T2DM, the presence of DS was associated with LDL-C, HDL-C levels and SBP, even after adjusting for sex, age, physical activity level and functional capacity. (Arq Bras Cardiol. 2020; 115(3):462-467)

## Introduction

Diabetes Mellitus, specifically Type 2 (T2DM) is a prevalent condition in the older adult population and is often accompanied by comorbidities and geriatric syndromes, including mental health-related illnesses, such as depression.^1^ Previous evidence has shown that the prevalence of depression is nearly twice as high in people with diabetes, and depression may also increase the risk of developing T2DM.^6,7^ In older adults with diabetes from low and middle-income countries, such as Brazil, this prevalence can reach 35.7%.^8^

In addition, older adults with T2DM have increased risk factors for cardiovascular disease (CVD), such as dyslipidemia, elevated blood pressure, impaired glycemic control and obesity.^9,10^ However, the clear role of T2DM in CVD may involve additional psychological factors, such as depression, which combined with diabetes may increase this risk. Previous findings have pointed to a relationship between the presence of depression and mostly lipid profile abnormalities in adults and/or older adults.^11^

Therefore, depression and CVD are common in patients with T2DM and have been linked to each other. However, the relationship between depressive symptoms (DS) and CVD in older adults with T2DM is understudied and often under-recognized.15 Thus, our goal is to examine the relationship between depressive symptoms and known cardiovascular risk factors in community-dwelling older adults with T2DM.

## Methods

### Sample and Study Design

This is a cross-sectional study carried out in a sample of community-dwelling older adults from the city of Recife, Brazil. The project was approved by the Institutional Human Research Ethics Committee of University of Pernambuco. All participants who agreed to participate in this study signed a clear and informed consent form.

The study participants were recruited as a convenience sample from 3,271 medical records of older patients (aged 60 years or more) with T2DM who visited the Geriatric and Endocrinology Clinics of a public university in Recife, Brazil. From these, 871 had a diagnosis of T2DM for more than 2 years. These subjects were contacted by telephone and invited to participate in the study. Thus, 198 participants returned the contact and volunteered to participate in the study, and were then referred for further eligibility screening.

The inclusion criteria were: having a medical diagnosis of T2DM for at least 2 years, and aged 60 years or older from both sexes. The exclusion criteria were: subjects using insulin, having cognitive impairment, neurological sequelae, severely decreased visual and/or hearing acuity, joint and/or muscle pain, lower limb amputations, wearing prostheses and/or presenting physical limitations that would limit their mobility.

After application of the eligibility criteria, the sample was reduced to 122 individuals. From these, 37 refused to undergo the blood test, leading to a final sample of 85 older adults with T2DM.

### Measures

Sociodemographic data (age and sex), clinical data (depressive symptomatology, functional capacity, and physical activity level), biochemical data and anthropometric measurements were recorded.

### Depressive Symptoms

Depressive symptoms were assessed using the Yesavage Geriatric Depression Scale - short version (GDS-15), which is validated for Brazilian older adults. Total score ranged from 0 to 15 points, where greater scores are related to the presence of depressive symptoms.^16^ Presence of depressive symptoms was dichotomized when the participants had 5 points or more;^16^ this categorization was used for descriptive purposes only. The total score was used in the multivariate analysis.

### Instrumental Activities of Daily Life assessment

Assessment of functional capacity was quantitatively analyzed based on the scores obtained in the Instrumental Activities of Daily Living (IADL).^17^ The presence of functional decline was seen in those patients who had complete or partial dependence on IADL.

### Physical Activity level

Physical activity level was derived from the self-reported time spent in moderate intensity activities (T_MIA_). This measure was calculated from the questions: “*During the last 7 days, on how many days did you perform moderate physical activities for at least 10 minutes at a time?” and “How much time did you usually spend doing moderate physical activities on one of those days?*” from the International Physical Activity Questionnaire. The T_MIA_ was calculated as follows = (*n days*) x (*time in min*).^18^

### Chronic Conditions and Medications

Presence of chronic conditions other than T2DM was registered as self-report by the interviewer, with participants being asked “Have you ever had a medical diagnosis of another chronic condition?”. If a positive answer was given the interviewer asked details about the disease. All participants had been on optimized drug therapy for T2DM for more than three months. The medication used by the older adults was delivered monthly by the Brazilian public health system during medical appointments, with the metformin being used as a hypoglycemic agent. Other medications in use were registered.

### Cardiovascular Risk Factors

Fasting plasma glucose (FPG) and lipid profile (serum triglycerides (TG), serum total cholesterol (TC), serum low-density lipoprotein cholesterol (LDL-C) and serum high-density lipoprotein cholesterol (HDL-C)) were collected from venous blood samples. Blood sample was drawn from an antecubital vein early in the morning, after a 12-hour fast, and assessed by a biochemical laboratory. The analyses were performed at the “Amaury de Medeiros” Integrated Health Center of the University of Pernambuco (CISAM/UPE). Measured parameters included: serum biochemistries performed by an automated enzymatic method, under routine laboratory procedures. The LDL-C was calculated using the Friedewald formula.^19^ The normal values for the parameters FPG, TG, TC, LDL-C, and HDL-C used in this research were defined by the revised National Cholesterol Education Program (NCEP) Adult Treatment Panel III (ATP III).^20^

Height (cm) was measured to the nearest 0.1 cm with a measuring tape attached to the wall. Body weight (kg) measures were obtained by using a calibrated scale with participants wearing light indoor clothes and no shoes. Body mass index was calculated using weight and height^2^ (kg/m^2^). Clinical assessment of blood pressure was performed with the patient in a seated position by the indirect auscultatory method using an aneroid sphygmomanometer.

### Statistical Analysis

Continuous variables are presented as mean and standard deviation, and categorical variables as absolute values and percentages. Data normality was tested by the Kolmogorov-Smirnov test. The normal probability plot and histogram for depressive symptom scores revealed a curvilinear line and positive skewed distribution. Therefore, depressive symptom scores were log10-transformed to improve linearity and normality. Associations between the presence of depressive symptoms and categorical variables were tested using the chi-square test. The analyses for continuous variables between groups were tested by the independent Student’s t-test.

Poisson multiple regression was performed to test the association of depressive symptoms with each CVD risk factor. Prevalence ratio (PR) estimates with the respective 95% CI were calculated. In order to avoid multicollinearity problems, each CVD risk factor and the depressive symptoms were analyzed separately in different models. All models were adjusted by sex, age, time spent in moderate physical activity, and functional status. These covariates were selected based on previous literature review.^21^ The significance level adopted for the analysis was 5%. Statistical analysis was performed using the Statistical Package for Social Science (SPSS), version 20.0.

## Results

The characteristics of the sample can be found in Table 1. Twenty-four of the eighty-five participants (28.2%) had depressive symptoms. There were no differences between sex and age among those participants with and without depressive symptomatology. However, those participants with depressive symptoms had lower IADL scores (23.63 ± 3.53; p-value= 0.02) and less time spent in moderate physical activities (28.63 ± 49.20; p-value=0.02) compared to those without depressive symptoms (Table 1).

**Table 1:** – General sample characteristics according to the presence of depressive symptoms

	Depressive symptoms			
Variables	All	No	Yes	p-value
	(n=85)	(n=61)	(n=24)	
Age (years), mean ± SD	70.59 (7.43)	69.33 (6.69)	71.08 (7.70)	0.33
Sex, n (%)				
*Male*	20 (23.5)	15 (25.0)	5 (20.8)	0.71
*Female*	65 (76.5)	46 (75.4)	19 (79.2)	
Self-rated health, n (%)				
*Reasonable/Poor*	76 (89.4)	53 (86.9)	23 (95.8)	0.43
*Excellent/Good*	9 (10.6)	1 (4.2)	8 (13.3)	
IADL score	24.99 (2.85)	25.52 (2.37)	23.63 (3.53)	0.02
BMI (kg/m^2^)^a^	28.63 (5.05)	28.90 (4.81)	27.96 (5.66)	0.45
Physical activity	48.22 (60.46)	56.78 (64.89)	28.63 (42.90)	0.03
Fasting plasma glucose (mg/dL)	159.01 (65.87)	153.75 (63.33)	172.38 (71.57)	0.24
Systolic blood pressure (mmHg)^d^	153.06 (80.85)	146.52 (25.69)	142.09 (20.41)	0.37
Diastolic blood pressure (mmHg)^d^	84.32 (15.83)	82.78 (19.72)	85.17 (14.17)	0.58
HDL-C (mg/dL)	58.53 (23.53)	61.25 (21.75)	52.83 (26.93)	0.16
LDL-C (mg/dL)	106.74 (41.35)	100.18 (33.13)	123.13 (54.31)	0.06
Total-C (mg/dL)	194.74 (47.65)	202.88 (58.22)	191.54 (42.92)	0.39
Triglycerides (mg/dL)	153.88 (80.85)	151.83 (87.82)	156.83 (62.86)	0.83

*IADL: Instrumental Activities of Daily Life; BMI: Body Mass Index;* ^*a*^ *4 missing values;* ^*b*^ *1 missing value;* ^*c*^ *6 missing values;* ^*d*^ *3 missing values.*

The most frequent chronic conditions reported in our sample were hypertension 10 (11.8%), followed by osteoarthrosis 4 (4.7%). Other conditions such as osteoporosis 1 (1.2%), bronchitis 1 (1.2%) and hypothyroidism 1 (1.2%) were also reported. Participants with hypertension diagnosis were in use of angiotensin-converting enzyme (ACE) inhibitors, beta-blockers or diuretics.

Table 2 shows the results of Poisson regression of the association between depressive symptoms and each CVD risk factor. After adjustments for sex, age, physical activity and functional capacity, high levels of LDL-C were related to increased DS (PR=1.005, CI 95% 1.002-1.008). HDL levels and low DS were also observed (PR=0.99, CI 95% 0.98-0.99). Besides, higher systolic blood pressure were associated to higher DS (PR=1.009, CI 95% 1.004-1.014) (Table 2).

**Table 2:** – Poisson multiple regression between depressive symptom scores and cardiovascular risk factors

	Depressive symptoms		
Cardiovascular risk factors	PR	CI 95%	p-value
BMI	0.98	0.96-1.01	0.47
Fasting plasma glucose (mg/dL)	1.001	0.99-1.00	0.32
Systolic blood pressure (mmhg)	1.009	1.004-1.014	<0.01
Diastolic blood pressure (mmHg)	1.00	0.99-1.00	0.42
HDL-C (mg/dL)	0.99	0.98-0.99	<0.01
LDL-C (mg/dL)	1.005	1.002-1.008	<0.01
Triglycerides (mg/dL)	1.00	0.99-1.00	0.61
Total-C (mg/dL)	1.00	0.99-1.00	0.19

*PR: Prevalence ratio, adjusted by sex, age, time spent in moderate physical activity, and functional status.*

## Discussion

In this study, we found that levels of LDL-C and HDL-C and systolic blood pressure were directly related to greater depressive symptoms in older adults with T2DM. This association was independent of sex, age, physical activity and functional capacity.

In prior general population studies, depressive symptoms were associated with CVD risk factors;^14,22^ however, studies on the association between T2DM and depressive symptoms in older adults are rare. Rice et al. (2010) found that higher levels of depressive symptoms in healthy older women were associated with greater LDL-C, which is in line with our results.^22^ Similarly, Liang et al. (2014) found that increased depressive symptoms in older Chinese adults were associated with high LDL-C levels.^14^ In a sample of 1,469 Brazilian community-dwelling older adults from the Bambuí Study, an association was found between abnormalities in the lipid profile and high depressive symptoms.^24^

A recent systematic review with meta-analysis found that lower rates of depression were related with lower serum LDL levels when modeling LDL as a categorical measure; however, low levels of LDL were present in depressive participants when considering it as a continuous measure, which is opposite to our findings.^13^ The authors discuss a temporal association between serum LDL and depression, where low levels of LDL may be related to the onset of depression. In turn, high levels of LDL could be a consequence of chronic depression, where the progression of the condition would lead to weight gain, and subsequent onset of the metabolic syndrome and high serum LDL.^13^

The relationship between those risk factors and the depressive symptoms remains controversial; some studies in the general elderly population found an association between depressive symptoms and risk factors for CVD,^22,25^ while others did not find any association between them. ^26^ We also found a significant association between other known CVD risk factors, such as high blood pressure and HDL levels and low depressive symptoms. In a study conducted in older Latino adults with diabetes, the presence of depressive symptoms was a risk factor for metabolic syndrome, low HDL-C levels and high levels of triglycerides.^23^ The relationship between higher systolic blood pressure and higher depressive symptoms was also seen in previous studies.^27,28^

Some pathways are linked to the relationship between depression and cardiometabolic dysregulation. Prior studies have shown that unhealthy lifestyle and the presence of inflammatory cytokines could be involved in this relationship.^29^ For instance, depressed people present increased smoking and alcohol consumption. In addition, they often have an unhealthy diet and engage in less physical activities.^29^ Moreover, depression may be related to weight gain (partly as a result of inactivity and unhealthy dietary choices), which promotes inflammation^29^ and is associated with decreased HDL and increased LDL levels.^30^ The association between depression and metabolic abnormalities may be bidirectional, and this co-occurrence can be particularly important in diabetic patients. In fact, there is evidence that it is not depression per se, but the co-occurrence of high depressive symptoms and cardiometabolic abnormalities that are related to increased risk of diabetes in older adults.^32^

It is important to mention the influence that some medications or clinical conditions may have on the relationship between depressive symptomatology and cardiovascular risk in older adults. For instance, some medications, such as antidepressives (e.g. fluoxetine), have been observed to induce lipid abnormalities.^33^ However, in our sample none of the participants were being treated with such medications. Therefore, our results were not influenced by this variable. In addition, some clinical conditions may increase the presence of depressive symptoms or cholesterol levels, as in cases of older adults with undiagnosed or untreated hypothyroidism.^34^However, in our sample, only one participant had a medical diagnosis of hypothyroidism, and this condition was controlled by medication.

This study presents some limitations that should be addressed. First, the small-sized, convenience sample hinders the generalization of the results and makes it problematic the adjustment by Poisson regression for other confounders (such as smoking, alcohol history or medication use).^29^ Furthermore, the cross-sectional nature does not allow us to distinguish whether the depressive symptoms preceded or followed the CVD outcomes. Finally, measures of depressive symptomatology were obtained by self-report, using the 15-item GDS, which is considered a screening tool rather than a clinical diagnostic tool. Nevertheless, it is a widely used instrument to identify individuals at high risk of depression.^16,35^

Despite these limitations, to the authors’ knowledge this is the first study to investigate the relationship between depressive symptoms and cardiovascular risk factors in a sample of older adults with diabetes. Despite the small magnitude of our results, our findings may provide useful insight about the importance of concomitantly including an assessment of psychological and cardiovascular health in older diabetic adults. Thus, this may represent an important group to focus on mental health and lifestyle interventions, to verify whether treating depression would lead to LDL cholesterol improvements, thus preventing adverse health outcomes. Additionally, future research should consider a longitudinal design to study the onset of depressive symptomatology in this population.

## Conclusion

In a sample of older adults with T2DM, the presence of depressive symptoms was associated to levels of LDL-C, HDL-C and systolic blood pressure even after adjusting for sex, age, physical activity level and functional capacity. More studies are needed to establish a temporal relationship between these two conditions in older adult populations with Type 2 Diabetes.
